# Supplementary material for: Molecular mechanism of activation-triggered subunit exchange in Ca2+/calmodulin-dependent protein kinase II
Source: eLife. 2016 Mar 7;5:e13405. doi: 10.7554/eLife.13405 (PMC4859805; doi:10.7554/eLife.13405)
Supplement: Supplementary file 1. — DOI: http://dx.doi.org/10.7554/eLife.13405.025 [file elife-13405-supp1.docx]

Supplemental Table 1

| **Data Collection** | | | | | | |
| --- | --- | --- | --- | --- | --- | --- |
|  | *S. rosetta* hub | *S. rosetta* kinase | *Nematostella*  CaMKII-B (pH 4.2) | | *Nematostella*  CaMKII-A (pH 7.0) | Human hub |
| Wavelength (Å) | 1.000 | 1.000 | 1.000 | | 1.000 | 1.000 |
| Space group | P6_1_22 | P4_3_ | P3_1_21 | | C222_1_ | P4_3_2_1_2 |
| Cell dimensions  (a,b,c) (Å) | 96.80, 96.80, 58.42 | 100.03, 100.03,  123.79 | 113.97, 113.97, 241.64 | | 78.47, 180.10, 179.51 | 89.89, 89.89, 226.58 |
| Resolution (Å) | 48.40-1.75  (1.78-1.75) | 46.58-2.90 (3.08-2.90) | 49.35-3.00  (3.13-3.00) | | 47.68-2.35  (2.42-2.35) | 48.64-2.75 (2.90-2.75) |
| Rsym (%) | 4.3 (78.3) | 10.3 (70.0) | 13.4 (137.3) | | 7.0 (72.9) | 7.9 (112.4) |
| *I/σ(I)* | 22.2 (1.7) | 8.4 (2.1) | 11.1 (1.5) | | 15.1 (1.5) | 14.4 (2.1) |
| *CC_1/2_* | 99.9 (66.3) | 99.3 (71.1) | 99.7 (52.8) | | 99.8 (67.5) | 94.6 (70.9) |
| Completeness (%) | 99.6 (99.9) | 100 (100) | 99.9 (100.0) | | 99.4 (95.2) | 100 (100) |
| Multiplicity | 5.1 (4.2) | 3.8 (3.8) | 5.5 (5.6) | | 6.1 (2.8) | 7.9 (7.6) |
| Wilson B factor | 23.6 | 51.72 | 39.0 | | 39.3 | 78.0 |
| **Refinement** | | | | | | |
| Resolution | 1.75 | 2.9 | 3.00 | 2.35 | | 2.75 |
| Reflections used | 16674 | 53172 | 37135 | 101711 | | 46067 |
| Rfree reflections | 853 | 2582 | 1816 | 4993 | | 2169 |
| ­R_work_/R_free_ | 19.13/21.11 | 18.26/22.43 | 22.71/26.51 | 19.24/21.98 | | 22.00/26.84 |
| Number of atoms | | | | | | |
| Protein | 1019 | 4870 | 8956 | 7300 | | 6297 |
| Ligands | 23 | 15 | 0 | 12 | | 0 |
| Average B factors | | | | | | |
| Protein | 33.3 | 85.9 | 105.8 | 55.6 | | 95.4 |
| Solvent | 43.3 | 65.5 | 85.3 | 51.2 | | 73.3 |
| Root mean square deviation from ideality | | | | | | |
| Bonds (Å) | 0.005 | 0.004 | 0.003 | 0.008 | | 0.003 |
| Angles (Å) | 0.913 | 0.609 | 0.598 | 0.847 | | 0.513 |
| Ramachandran Statictics | | | | | | |
| Favored (%) | 96.9 | 94.2 | 93.6 | 97.8 | | 97.1 |
| Disallowed (%) | 0.0 | 0.3 | 0.9 | 0.0 | | 0.1 |
| MolProbity clash score | 3.40 | 6.55 | 7.43 | 3.04 | | 5.51 |
